# Antibodies Are Required for Complete Vaccine-Induced Protection against Herpes Simplex Virus 2

**DOI:** 10.1371/journal.pone.0145228

**Published:** 2015-12-15

**Authors:** William P. Halford, Joshua Geltz, Ronald J. Messer, Kim J. Hasenkrug

**Affiliations:** 1 Dept of Microbiology and Immunology, Southern Illinois University School of Medicine, Springfield, IL, 62702, United States of America; 2 Laboratory of Persistent Viral Diseases, Rocky Mountain Laboratories, National Institute of Allergy and Infectious Diseases, National Institutes of Health, Hamilton, MT, 59840, United States of America; University of Cincinnati School of Medicine, UNITED STATES

## Abstract

Herpes simplex virus 2 (HSV-2) 0ΔNLS is a live HSV-2 *ICP0* ^-^ mutant vaccine strain that is profoundly attenuated *in vivo* due to its interferon-hypersensitivity. Recipients of the HSV-2 0ΔNLS vaccine are resistant to high-dose HSV-2 challenge as evidenced by profound reductions in challenge virus spread, shedding, disease and mortality. In the current study, we investigated the requirements for HSV-2 0ΔNLS vaccine-induced protection. Studies using (UV)-inactivated HSV-2 0ΔNLS revealed that self-limited replication of the attenuated virus was required for effective protection from vaginal or ocular HSV-2 challenge. Diminished antibody responses in recipients of the UV-killed HSV-2 vaccine suggested that antibodies might be playing a critical role in early protection. This hypothesis was investigated in B-cell-deficient μMT mice. Vaccination with live HSV-2 0ΔNLS induced equivalent CD8^+^ T cell responses in wild-type and μMT mice. Vaccinated μMT mice shed ~40-fold more infectious HSV-2 at 24 hours post-challenge relative to vaccinated wild-type (B-cell^+^) mice, and most vaccinated μMT mice eventually succumbed to a slowly progressing HSV-2 challenge. Importantly, passive transfer of HSV-2 antiserum restored full protection to HSV-2 0ΔNLS-vaccinated μMT mice. The results demonstrate that B cells are required for complete vaccine-induced protection against HSV-2, and indicate that virus-specific antibodies are the dominant mediators of early vaccine-induced protection against HSV-2.

## Introduction

Herpes simplex virus type 2 (HSV-2) is one of the most common sexually transmitted infections. Worldwide, over 500 million people between the ages of 14 and 49 are infected [1]. HSV-2 is an α-herpesvirus that persists for life and is periodically shed, often asymptomatically. Carriers may shed HSV-2 in their genital tract in the absence of lesions [2, 3] and more than 10 million people are newly infected with HSV-2 each year. HSV-2 is the primary cause of recurrent genital herpes, and HSV-2 carriers have a 3-fold higher risk of acquiring HIV [4–6]. Mother-to-newborn transmission of HSV-2 occurs in about 1 per 10,000 live births, and often progresses to the devastating disease of neonatal herpes [7–10]. Antiviral drugs reduce, but do not eliminate, these risks. For all of these reasons, it is widely agreed that an effective HSV-2 vaccine is needed.

The majority of successful viral vaccines have been based upon live-attenuated variants of the wild-type virus. This includes childhood vaccines for mumps, measles, rubella and varicella-zoster virus (VZV). Like HSV-2, VZV is an α-herpesvirus that causes a primary infection (chickenpox), establishes a latent infection in the peripheral nervous system, and may later reactivate to cause disease (shingles). The live-attenuated VZV Oka vaccine has proven safe and effective [11, 12], and this raises the possibility that a live-attenuated HSV-2 virus may likewise be adequate to stop the spread of HSV-2 genital herpes.

We have previously described a live-attenuated HSV-2 vaccine, HSV-2 0ΔNLS, which contains an in-frame deletion in the *ICP0* gene. HSV-2's ICP0 protein is an immediate-early co-activator of viral mRNA synthesis [13, 14, 15], and functions as a master regulator of HSV's latency-replication balance [16, 17]. The HSV-2 0ΔNLS vaccine strain contains an in-frame deletion that removes ICP0's nuclear localization signal (0ΔNLS), and thus prevents ICP0 from serving as a co-activator of viral mRNA synthesis. In the absence of full ICP0 function, HSV-1 and HSV-2 *ICP0* ^-^ mutant viruses are hypersensitive to type I interferon [18] and are profoundly attenuated in lymphocyte-deficient *rag2* ^-/-^ mice [15, 19]. In vaccinated animals, the HSV-2 0ΔNLS vaccine strain undergoes limited replication at the immunization site, but fails to sustain replication long enough to cause pathogenesis [15]. Importantly, the HSV-2 0ΔNLS vaccine elicits an adaptive immune response that protects mice against lethal challenge with 1,000 times the LD_50_ of wild-type HSV-2 [20, 21]. Thus, this is a safe and highly effective vaccine in mice.

One feature of live-attenuated viruses that may contribute to their efficacy is a high degree of antigenic breadth. A broad range of viral antigens allows individuals of different MHC types to mount a protective immune response. Because each arm of the adaptive immune response engages different epitopes, a live vaccine offers the greatest chance of eliciting polyclonal responses that include a diverse population of effector B cells, helper T cells, and cytolytic T cells. The live HSV-2 0ΔNLS vaccine retains 99.3% of HSV-2's protein-coding capacity, and encodes 70 viral proteins that may contribute to the ensuing adaptive immune response. In contrast, most HSV-2 vaccines tested in clinical trials introduce recipients to 1 to 3% of HSV-2's proteome in the form of glycoprotein D and one or two other HSV-2 proteins. Specific examples include Glaxo Smith Kline's Herpevac vaccine [22, 23], Genocea's GEN-003 vaccine [24], Vical's HSV-2 vaccine [25, 26], and Coridon's HSV-2 vaccine [27].

In side-by-side comparisons, the live HSV-2 0ΔNLS vaccine is more effective than an HSV-2 glycoprotein D subunit vaccine similar in formulation to Glaxo Smith Kline's Herpevac vaccine [20, 21]. Mice or guinea pigs immunized with the live HSV-2 0ΔNLS vaccine produce ~40-fold higher levels of total HSV-2-specific antibody relative to animals immunized with a glycoprotein D vaccine. Likewise, recipients of the live HSV-2 0ΔNLS vaccine are up to 100-times better protected against HSV-2 vaginal challenge than animals immunized with a glycoprotein D vaccine [20, 21]. We have advanced a hypothesis that the superior efficacy of the HSV-2 0ΔNLS vaccine may, at least in part, be due to its 100-fold increase in antigenic breadth relative to glycoprotein D-based vaccines [28]. Consistent with this possibility, animals immunized with the live HSV-2 0ΔNLS vaccine mount an antibody response against 9 to 19 different viral proteins [29].

In the current study we investigated the relative protection from the live HSV-2 0ΔNLS vaccine relative to a 'killed' (inactivated) vaccine, and examined the requirement for B-cell responses in protection from virus challenge. The results indicated that effective vaccination with the HSV-2 0ΔNLS vaccine requires replication of the live vaccine in recipients, and that T- and B-cell responses were both required for complete vaccine-induced protection against HSV-2.

## Results

### Effect of UV-inactivation on infectivity and antigen expression from HSV-2 0ΔNLS

**Fig 1:**
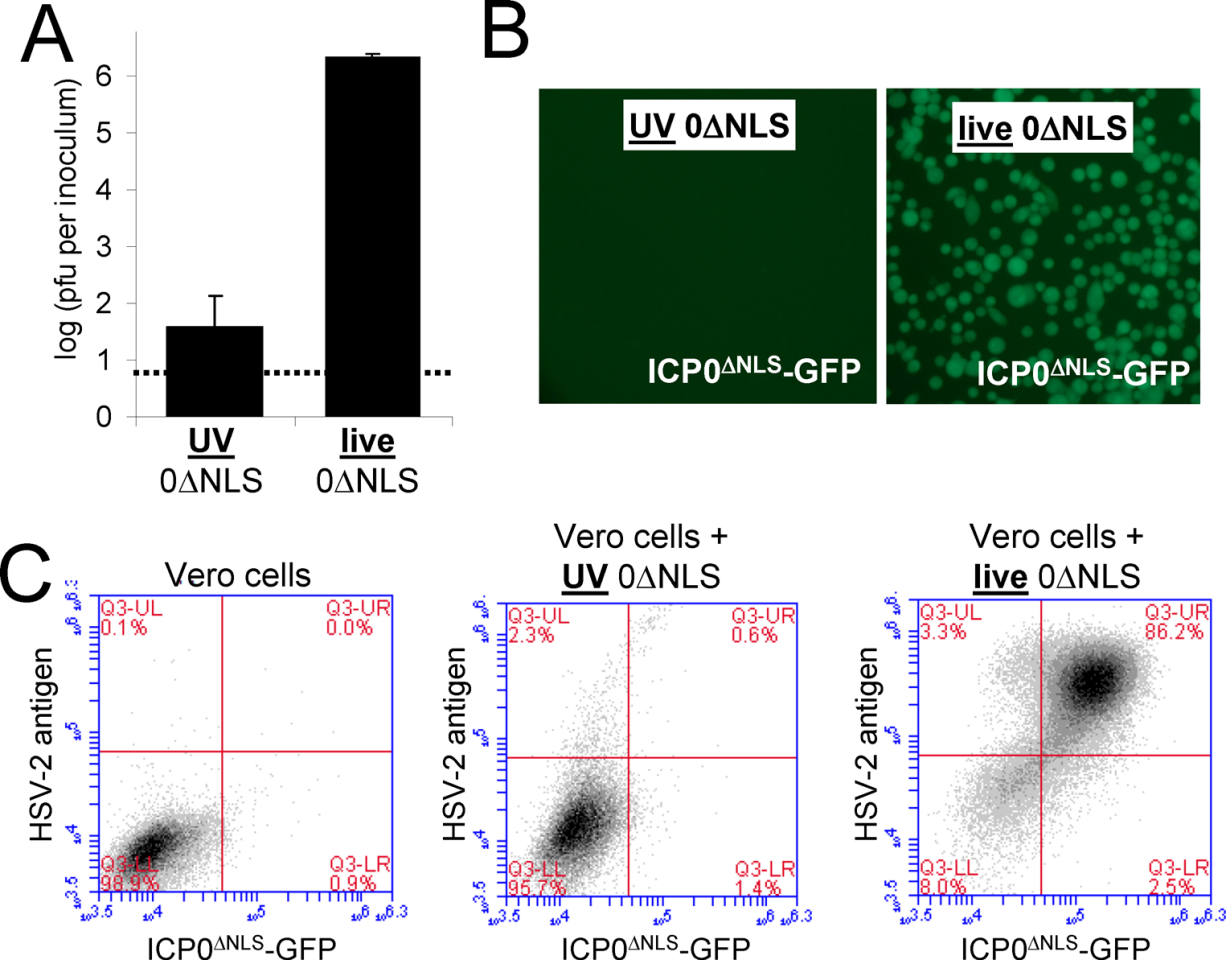
UV-inactivation of HSV-2 0ΔNLS ablates *de novo* synthesis of viral antigens. **(A)** Effect of UV-inactivation on infectivity of HSV-2 0ΔNLS, as determined by plaque assay. The dashed line denotes the lower limit of detection of the plaque assay. **(B and C)** Capacity of live-0ΔNLS vaccine (MOI = 10) versus an equivalent amount of UV-0ΔNLS vaccine to mediate *de novo* protein synthesis in Vero cells was evaluated at 18 hours post-inoculation by **(B)** fluorescence microscopy of the mutant ICP0^ΔNLS^-GFP protein and **(C)** flow cytometric analysis of ICP0^ΔNLS^-GFP versus total HSV-2 antigen. In the latter test, total HSV-2 antigen was stained with rabbit polyclonal anti-HSV antibody and APC-goat anti-rabbit IgG.

UV irradiation reduced the infectivity of HSV-2 0ΔNLS by ~50,000-fold, and destroyed the capacity of HSV-2 0ΔNLS to express its GFP-tagged ICP0^ΔNLS^ protein (Fig 1A and 1B). Flow cytometric analysis verified that Vero cells exposed to UV-inactivated HSV-2 0ΔNLS did not express the GFP-tagged, mutant ICP0^ΔNLS^ protein and contained only modest levels of total HSV-2 antigen (Fig 1C). In contrast, Vero cells inoculated with live HSV-2 0ΔNLS expressed the GFP-tagged, mutant ICP0^ΔNLS^ protein and total HSV-2 antigen to levels that were ~30-fold above background at 18 hours post-inoculation (Fig 1C). Subsequent experiments focused on determining if UV-inactivation of HSV-2 0ΔNLS affected the immunogenicity or protective efficacy of this vaccine.

### Both live-0ΔNLS and UV-0ΔNLS vaccines induce CD8^+^ T cell responses

**Fig 2:**
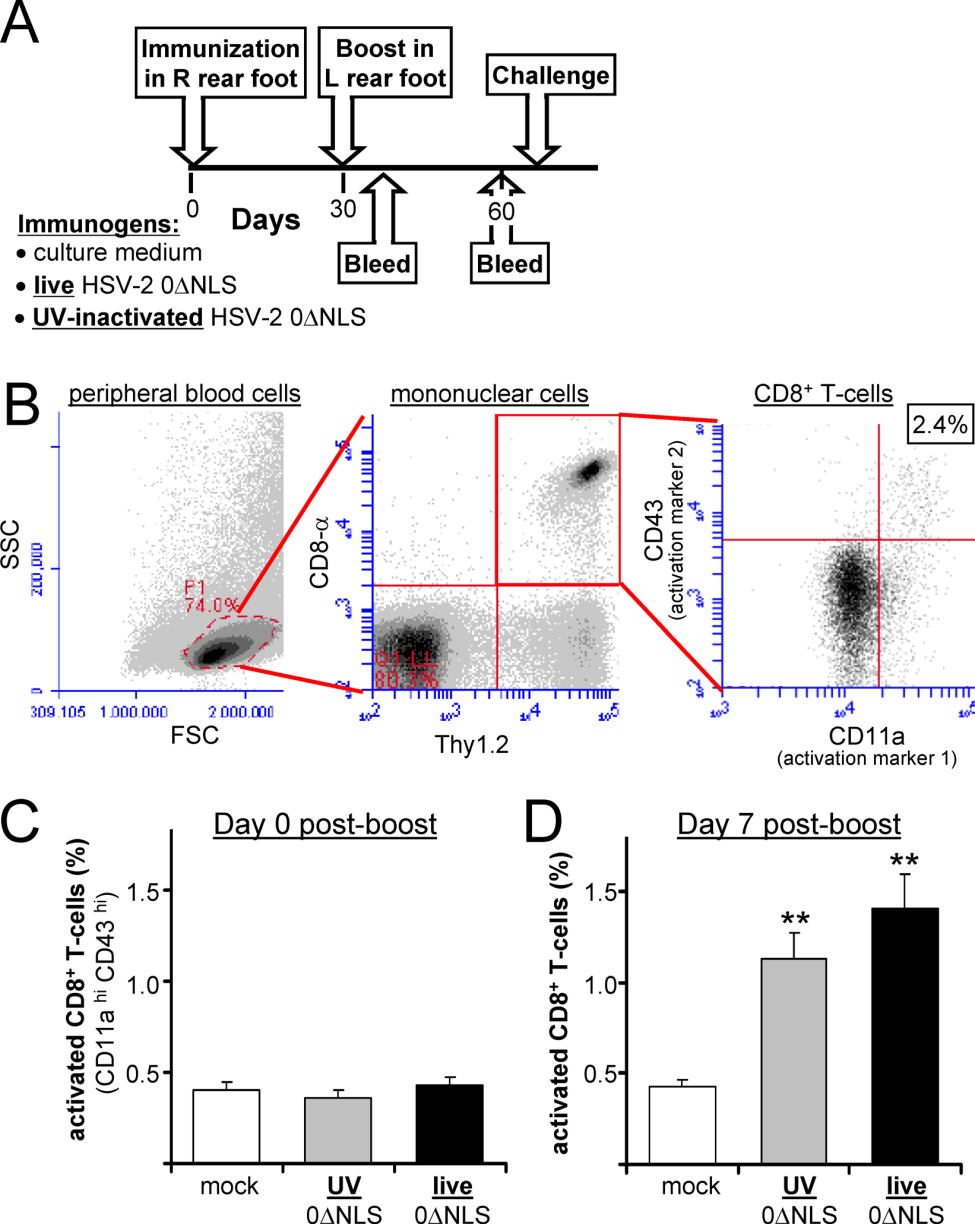
Live 0ΔNLS and UV-0ΔNLS vaccines activate peripheral CD8^+^ T-cells at a comparable frequency in C57BL/10 mice. **(A)** Design of vaccine-challenge studies. Mice received footpad immunizations on Days 0 and 30 with culture medium (mock), U.V-0ΔNLS, or 2 x 10^6^ pfu live 0ΔNLS, and blood was collected on Day 7 post-boost to analyze CD11a and CD43 activation marker expression on CD8^+^ T-cells. **(B)** Within peripheral WBCs, a primary gate was set on cells with the forward- and side-scatter properties of mononuclear cells, and a secondary gate was set on CD8^+^ Thy1.2^+^ T-cells, which were analyzed for the frequency of cells expressing elevated levels of CD11a and an activation-associated glycoform of CD43. **(C and D)** Mean ± sem frequency of CD11a^hi^, CD43^hi^ CD8^+^ T-cells in peripheral blood of mice on **(C)** Day 0 and **(D)** Day 7 post-boost (n = 16 per group per time). A double asterisk (**) denotes p < 0.001 that the frequency of CD11a^hi^, CD43^hi^ CD8^+^ T-cells was equivalent to mock-immunized control mice on that day, as determined by one-way ANOVA and Tukey's post-hoc-test.

C57BL/10 mice were immunized on Days 0 and 30 in their right and left rear footpads, respectively, with equivalent volumes of *i*. culture medium, *ii*. ultraviolet (UV)-inactivated HSV-2 0ΔNLS (UV-0ΔNLS), or *iii*. live HSV-2 0ΔNLS (live-0ΔNLS) (Fig 2A). As has been established in other models [30, 31], a non-lethal assay was developed to gauge the total CD8^+^ T-cell response to the HSV-2 0ΔNLS vaccine by measuring the frequency of CD8^+^ T cells that expressed elevated levels of the CD11a adhesion molecule [32, 33] and an activation-associated glycoform of CD43 [34–36]. At Day 7 post-boost, mice were bled and peripheral WBCs were enriched using Dextran T-500 and ammonium chloride lysis of RBCs. Flow cytometry was used to gate on peripheral WBCs that were Thy1.2^+^ CD8^+^ T cells, and the frequency of CD8^+^ T cells exhibiting a CD11a^hi^, CD43^hi^ phenotype was analyzed (Fig 2B). On Day 0 post-boost, all groups of mice exhibited the same baseline frequency (0.4 ± 0.1%) of CD8^+^ T cells that were CD11a^hi^, CD43^hi^ (Fig 2C). In contrast, on Day 7 post-boost, there was a significant increase in activated CD8^+^ T cells in groups immunized with either UV-0ΔNLS or live-0ΔNLS compared to mock (Fig 2D). Thus, both live- and UV-0ΔNLS vaccines activated similar frequencies of CD8^+^ T cells in wild-type C57BL/10 mice.

Parallel ELISpot analyses suggested that at least a subset of these activated CD8^+^ T cells were HSV-2-specific, as several HSV-2-specific T-cell epitopes identified by St. Leger, et al (2011) [37] stimulated at least a 2-fold increase in IFN-γ-spot-forming cells from splenocytes of live 0ΔNLS-vaccinated mice at Day 7 post-boost relative to an irrelevant ovalbumin (SIINFEKL) peptide. Specifically, the following HSV-2 peptides elicited significant IFN-γ-secretion from splenocytes of live 0ΔNLS-vaccinated mice: HSV-2 gB 498–505 (SSIEFARL); HSV-2 gB 452–460 (YQPLLSNTL); HSV-2 RR1 982–989 (FAPLFTNL); HSV-2 ICP8 219–237 (RSIGENFNYPLPFFNRPLA); and HSV-2 ICP8 773–791 (VKSRVLFAGASANASEAAK) (data not shown). In contrast, the same HSV-2 peptides failed to elicit a significant increase in IFN-γ-spot-forming cells from splenocytes of mock-vaccinated mice.

### Live-0ΔNLS vaccine induces better antibody responses than UV-0ΔNLS vaccine

**Fig 3:**
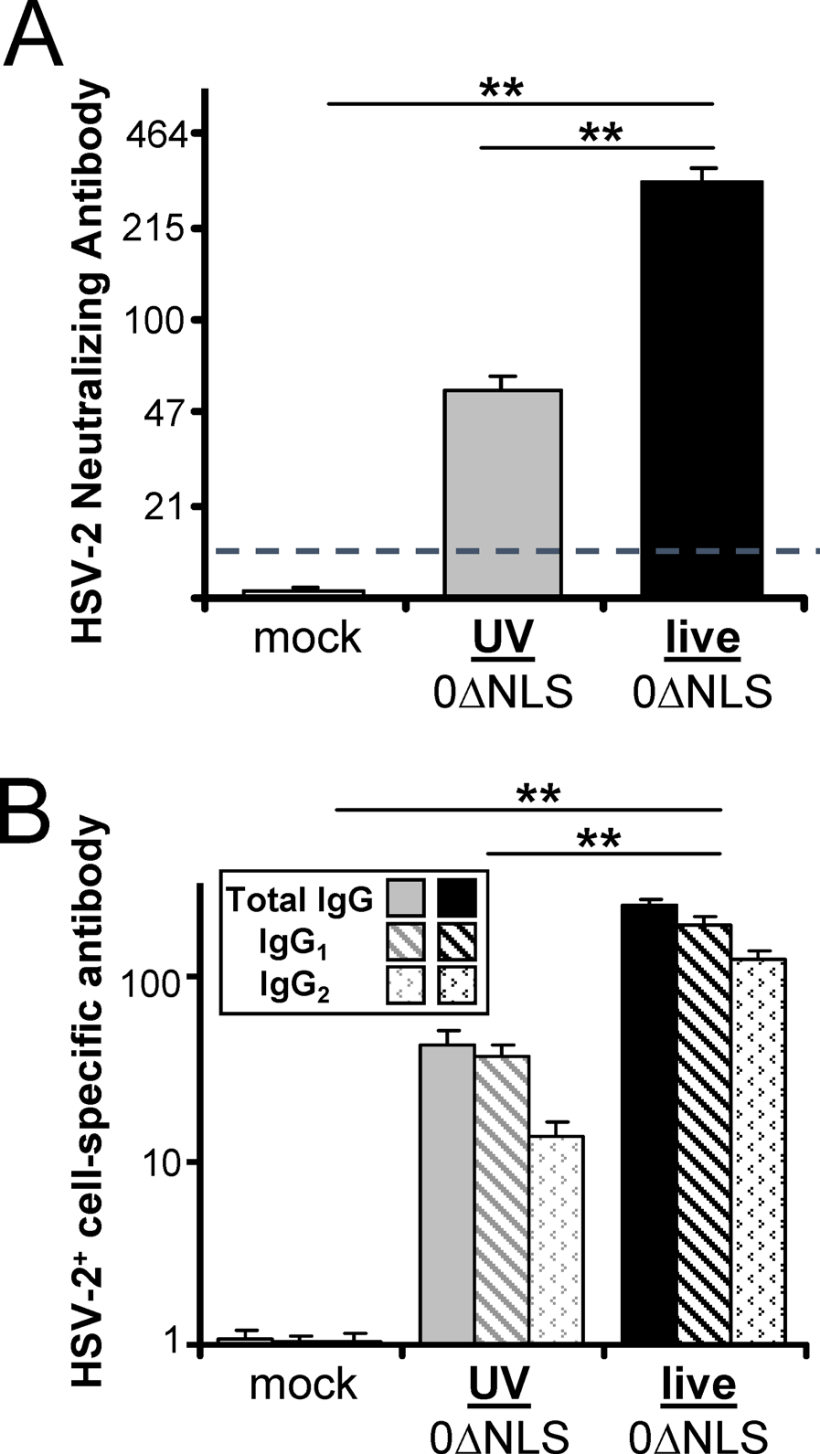
Live-0ΔNLS vaccine elicits a stronger IgG antibody response than UV-0ΔNLS vaccine. **(A)** Mean ± sem neutralizing antibody titer in pre-challenge (Day 60) serum collected from C57BL/10 mice immunized with mock versus UV-0ΔNLS or live-0ΔNLS vaccines (n = 16 per group). **(B)** Mean ± sem total HSV-2-specific IgG levels in pre-challenge serum, as determined by a flow cytometry-based ABVIC (antibody-binding to virus-infected cell) assay comparing the mean fluorescent intensity of IgG antibody bound to uninfected versus HSV-2-infected test cells (n = 16 per group). Mouse antibody-labeled test cells were secondarily labeled with APC-conjugated antibody specific for total IgG, IgG subclass 1, or IgG subclass 2. Double asterisks (**) denote p < 0.001 that neutralizing antibody or HSV-2^+^ cell-specific antibody levels were equivalent between live 0ΔNLS and UV-0ΔNLS-immunized mice, or mock-immunized mice, as determined by one-way ANOVA and Tukey's post-hoc-test.

On Day 60 post-vaccination, blood was collected and sera were tested for their capacity to neutralize the infectivity of HSV-2 virions. Mice immunized with the UV-0ΔNLS vaccine had neutralizing antibody titers of 55 ± 5, whereas mice immunized with the live-0ΔNLS vaccine had significantly higher neutralizing antibody titers of 310 ± 30 (Fig 3A). As a second measure of virus-specific antibody, flow cytometry was used to assess antibody-binding to HSV-2-infected cells (ABVIC) as previously described [20] (Fig 3B). Virus-specific total IgG was analyzed, as well as the IgG_1_ and IgG_2_ isotypes. The ABVIC assay showed that mice immunized with the live-0ΔNLS vaccine produced 5-fold higher titers of HSV-2-specific total IgG relative to UV-0ΔNLS-immunized mice (Fig 3B). Focusing on the potentially more protective IgG_2_ subclass of antibodies [38], the live-0ΔNLS vaccine elicited ~10-fold higher levels of HSV-2-specific IgG_2_ antibodies compared to those elicited by the UV-0ΔNLS vaccine (Fig 3B). These data indicated that the live-0ΔNLS vaccine was significantly better than the UV-0ΔNLS vaccine at eliciting antibodies that neutralized HSV-2 virions and bound HSV-2-infected cells.

### Ocular challenge of mice immunized with UV- versus live-0ΔNLS vaccines

**Fig 4:**
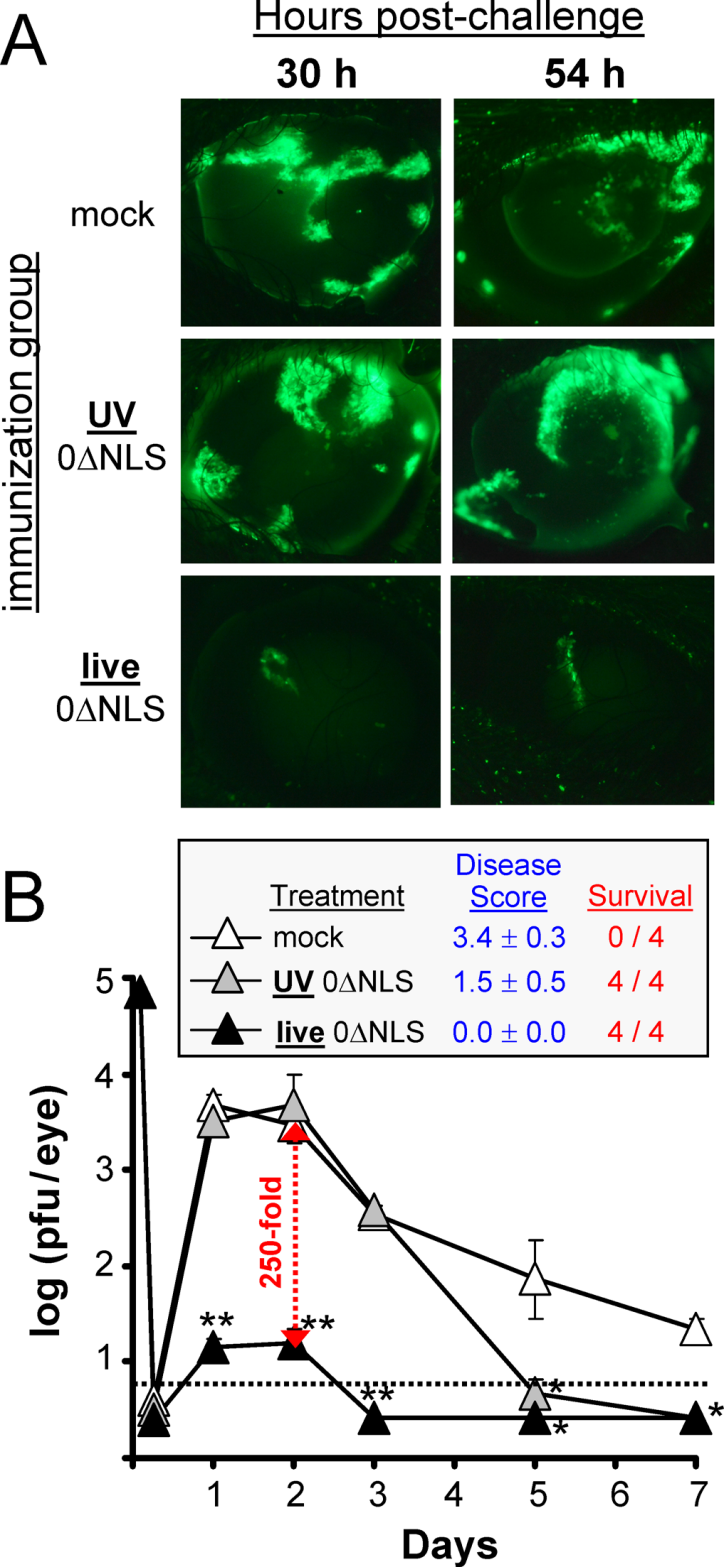
Ocular HSV-2 MS-GFP challenge of C57BL/10 mice immunized with UV-0ΔNLS or live-0ΔNLS vaccines. On Day 70, mock-, UV-0ΔNLS-, or live-0ΔNLS-immunized C57BL/10 mice were challenged with 70,000 pfu per eye of HSV-2 MS-GFP. **(A)** GFP expression showing the extent of HSV-2 MS-GFP spread in representative corneas of mice at 30 and 54 h post-challenge. **(B)** HSV-2 MS-GFP shedding from mouse eyes at times post-challenge (n = 4 per group). The dashed line denotes the lower limit of detection of the plaque assay. A single asterisk (*) denotes p < 0.05 and a double asterisk (**) denotes p < 0.001 that HSV-2 MS-GFP shedding was equivalent to mock-immunized control mice on that day, as determined by one-way ANOVA and Tukey's post-hoc-test.

The capacity of the live- versus UV-0ΔNLS vaccine to protect recipients against a lethal HSV-2 ocular challenge was compared. On Day 70 post-vaccination, mice were challenged with 70,000 pfu per eye of HSV-2 MS-GFP. At 30 and 54 hours post-challenge, mice were anaesthetized and their corneas photographed to visualize the extent of MS-GFP spread (Fig 4A). The corneas of mice immunized with the UV-0ΔNLS vaccine were similarly vulnerable to HSV-2 MS-GFP challenge relative to mock-immunized mice, as both groups exhibited high levels of GFP expression at 30 and 54 hours post-challenge (Fig 4A). In contrast, mice immunized with the live-0ΔNLS vaccine exhibited restricted GFP expression in their corneas, which was indicative of decreased virus spread (Fig 4A).

As a quantitative measure of viral infection, shedding of infectious HSV-2 MS-GFP into tears was assayed. During the first 72 hours post-challenge, mice immunized with UV-0ΔNLS shed high levels of virus that were equivalent to mock-immunized mice (Fig 4B). In contrast, mice immunized with the live-0ΔNLS vaccine shed an average 250-fold less virus during the first 72 hours post-challenge (Fig 4B). All live-0ΔNLS-immunized mice remained without clinical signs for 30 days post-challenge, whereas all mock-immunized mice developed lethal disease by Day 9. All UV-0ΔNLS-immunized mice survived HSV-2 MS-GFP challenge but exhibited frank periocular disease by Day 8 (Fig 4B). Thus, the live-0ΔNLS vaccine elicited robust protection against ocular HSV-2 challenge, which was significantly better than that elicited by the UV-0ΔNLS vaccine.

### Vaginal challenge of mice immunized with UV- versus live-0ΔNLS vaccines

**Fig 5:**
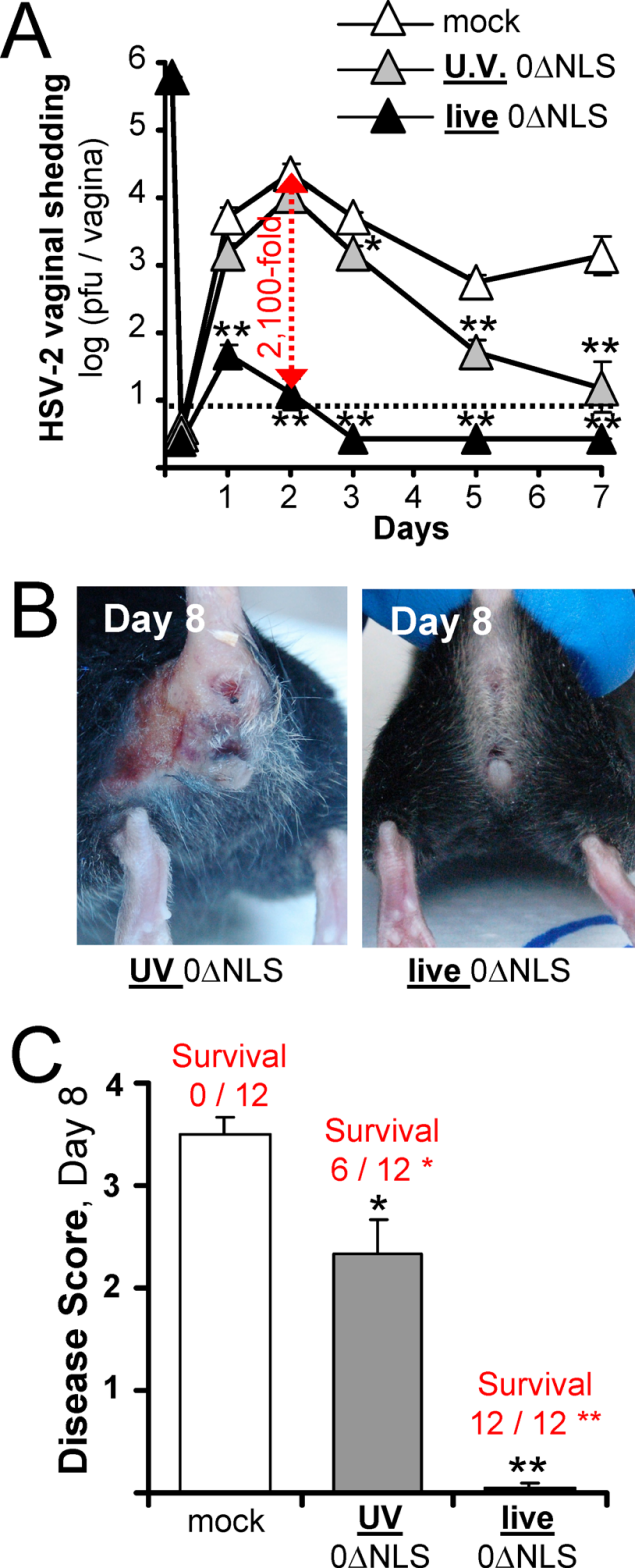
Vaginal HSV-2 MS challenge of C57BL/10 mice immunized with a UV-0ΔNLS vaccine or a live-0ΔNLS vaccine. Mice were treated with 2 mg medoxyprogesterone 7 and 3 days prior to vaginal HSV-2 challenge. On Day 70, mock-, UV-0ΔNLS-, or live-0ΔNLS-immunized C57BL/10 mice were challenged with 500,000 pfu per vagina of HSV-2 MS. **(A)** Mean ± sem HSV-2 MS shedding from mouse vaginas at times post-challenge (n = 12 per group). The dashed line denotes the lower limit of detection of the plaque assay. A single asterisk (*) denotes p < 0.05 and a double asterisk (**) denotes p < 0.001 that HSV-2 MS shedding was equivalent to mock-immunized control mice on that day, as determined by one-way ANOVA and Tukey's post-hoc-test. **(B)** Representative examples of perivaginal disease on Day 8 post-challenge in mice immunized with the UV-0ΔNLS or live-0ΔNLS vaccines. **(C)** Mean ± sem of disease scores in mice on Day 8 post-challenge. Regarding survival frequency, a single asterisk (*) denotes p < 0.05 and a double asterisk (**) denotes p < 0.001 that the frequency of survival was equivalent to mock-immunized control mice, as determined by Fisher's Exact Test.

On Day 70 post-vaccination, protection against intravaginal challenge with 500,000 pfu of wild-type HSV-2 MS was tested. During the first 72 hours post-challenge, UV-0ΔNLS-immunized mice shed high titers of HSV-2 MS that were only slightly lower than that shed by mock-immunized mice (Fig 5A). In contrast, live-0ΔNLS-immunized mice shed ~2,000-fold less HSV-2 MS per vagina at 48 and 72 hours post-challenge (Fig 5A). All UV-0ΔNLS-immunized mice exhibited mild to severe perivaginal disease and only half survived challenge (Fig 5B and 5C). In marked contrast, there was no overt disease in any live-0ΔNLS-immunized mice and all twelve animals survived wild-type HSV-2 challenge (Fig 5B and 5C). Thus the live-0ΔNLS vaccine was highly protective against high-dose challenge by both ocular and vaginal routes of infection.

### B-cell-deficient and wild-type mice mount CD8^+^ T-cell responses to the live-0ΔNLS vaccine

**Fig 6:**
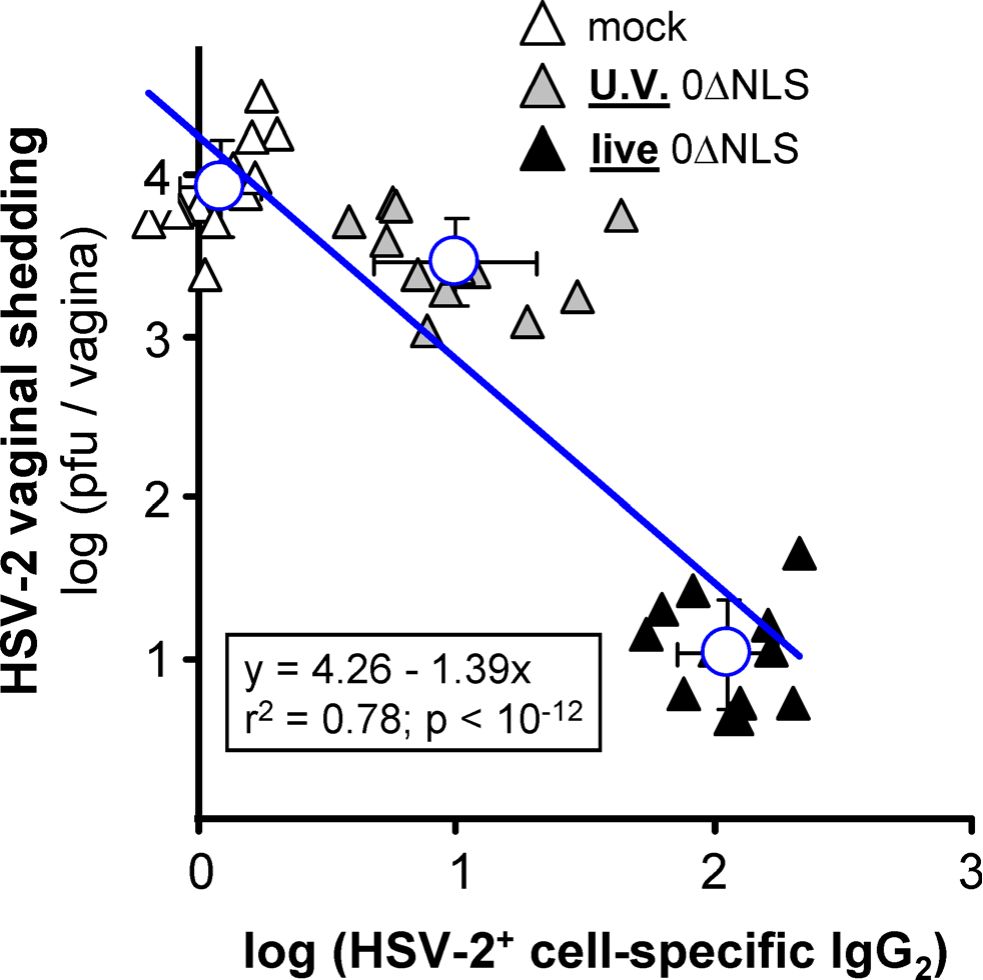
HSV-2^+^ cell-specific IgG_2_ levels correlate with protection against vaginal HSV-2 challenge in mice. C57BL/10 mice were immunized in their right and left, rear footpads on Days 0 and 30, respectively, with mock-, UV-0ΔNLS-, or live-0ΔNLS vaccines (n = 12 per group). On Day 60, blood was collected from all mice and HSV-2-specific IgG subclass 2 levels were analyzed by the ABVIC assay presented in Fig 3B. On Day 70, mice were vaginally challenged with wild-type HSV-2 MS as presented in Fig 5A. For each mouse (one symbol per animal), the average log (pfu/vagina) shed on Days 1, 2, and 3 post challenge (y-axis) was plotted as a function of log (HSV-2^+^ cell-specific IgG_2_) observed in the same mouse prior to challenge (x-axis). The solid blue line represents the best-fit linear regression model, y = 4.26–1.39x, for the 36 matched datum pairs and the goodness-of-fit for this slope (correlation) was 0.78 and was considered significant (p < 10^−12^). The mean ± sd of log (HSV-2^+^ cell-specific IgG_2_) for each immunization group is plotted as open blue circles.

We have previously shown that vaccine-induced, HSV-2^+^ cell-specific IgG antibody responses correlate with functional protection against HSV-2 challenge [20]. Likewise, in the current study, pre-challenge HSV-2^+^ cell-specific IgG_2_ antibody levels observed in mock-, UV-0ΔNLS-, or live 0ΔNLS-vaccinated animals strongly correlated with observed reductions in HSV-2 vaginal shedding in the same animals between Days 1 and 3 post-challenge (Fig 6; r^2^ = 0.78; p < 10^−12^). Thus, weaker antibody responses elicited by the UV-0ΔNLS vaccine correlated with weaker protection against HSV-2 challenge (Fig 6). This observation suggested that antibodies might be playing a direct effector role in protective immunity to HSV-2 challenge.

To test this hypothesis, we compared the efficacy of the UV-0ΔNLS versus live-0ΔNLS vaccines in C57BL/10 mice that were wild-type or B-cell-deficient (μMT). In some cases, B-cell deficiency has been reported to affect T-cell responses [39–41] while in other cases it has not [42]. To address this issue in our model, experiments were conducted to determine if the UV- or live-HSV-2 0ΔNLS vaccines elicited similar T-cell responses in wild-type versus μMT mice.

**Fig 7:**
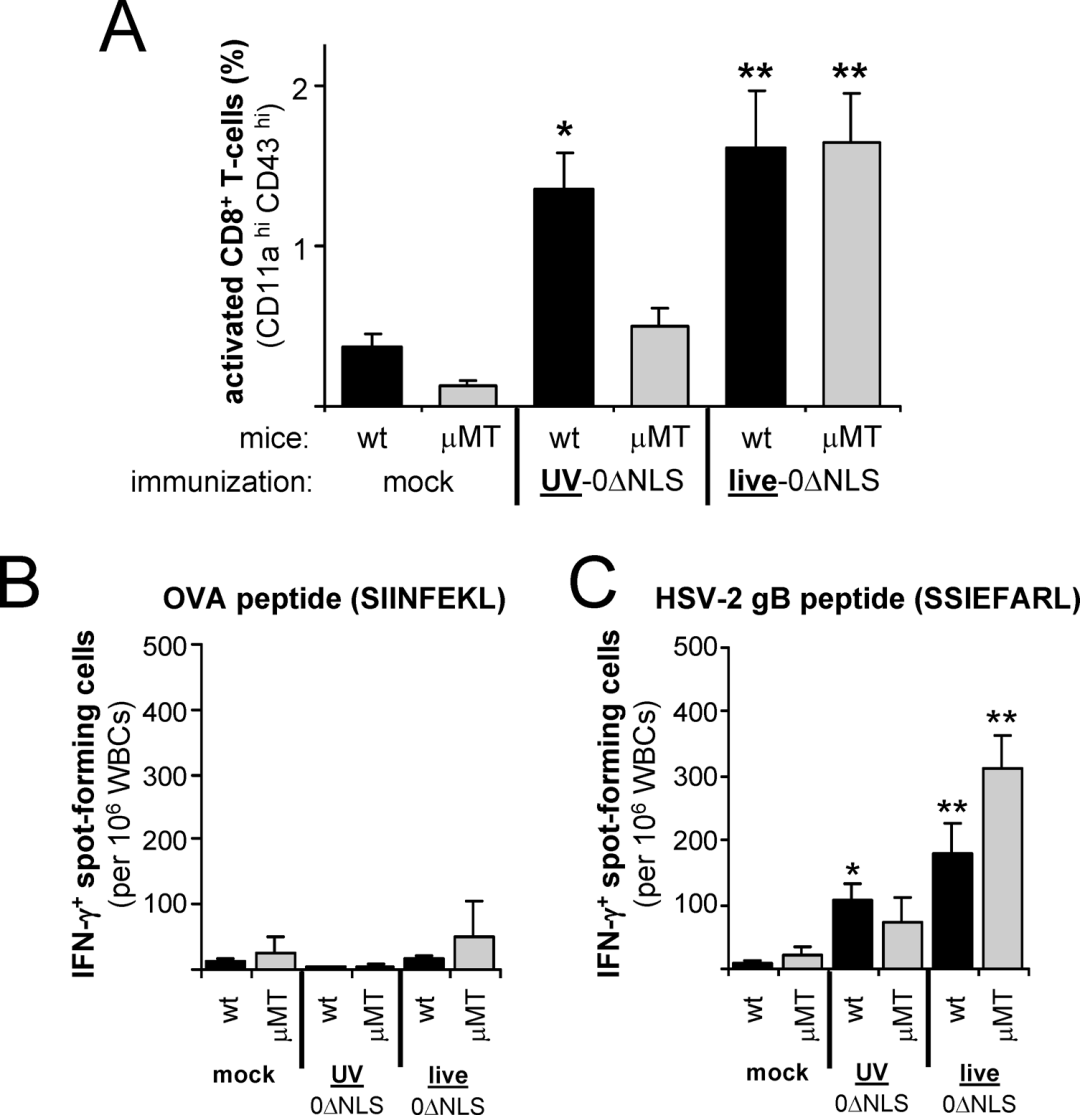
The live-0ΔNLS vaccine elicits comparable CD8^+^ T-cell responses in wild-type and μMT mice. C57BL/10 wild-type (wt) mice and μMT mice received footpad immunizations on Days 0 and 30 with culture medium (mock), U.V-0ΔNLS, or 2 x 10^6^ pfu live-0ΔNLS, and blood was collected on Day 7 post-boost to analyze CD11a and CD43 activation marker expression on CD8^+^ T-cells. **(A)** Mean ± sem frequency of CD11a^hi^, CD43^hi^ CD8^+^ T-cells in peripheral blood (n = 7 per group). **(B and C)** Spleen WBCs were also harvested from mice on Day 7 post-boost to compare T-cell activation by IFN-γ ELISpot. Mean ± sem number of IFN-γ^+^ spot-forming cells per million WBCs stimulated with **(B)** SIINFEKL (ovalbumin) peptide or **(C)** SSIEFARL (HSV-2 gB) peptide (n = 4 per group). In each panel, a single asterisk (*) denotes p < 0.05 and a double asterisk (**) denotes p < 0.001 that the observed value was equivalent to mock-immunized wild-type mice, as determined by one-way ANOVA and Tukey's post-hoc-test.

Wild-type and μMT mice were immunized on Days 0 and 30 in their right and left rear footpads, and CD8^+^ T-cell responses were analyzed at Day 7 post-boost. Immunization with the live-0ΔNLS vaccine elicited comparable frequencies of activated (CD11a^hi^ CD43^hi^) CD8^+^ T cells in wild-type and μMT mice (Fig 7A). In contrast, the UV-0ΔNLS vaccine elicited significantly weaker T-cell responses in μMT mice relative to wild-type mice (Fig 7A). IFN-γ ELISpot assays were used to verify that vaccine-induced CD8^+^ T-cell responses were HSV-2-specific. ELISpot assays were conducted with splenocytes from immunized mice incubated with an irrelevant ovalbumin peptide, SIINFEKL (Fig 7B), or the immunodominant HSV-2 gB_498-505_ epitope, SSIEFARL (Fig 7C; Ref. [37, 43]). Immunization with the live-0ΔNLS vaccine elicited strong gB-specific T-cell responses in both wild-type and μMT mice. In contrast, the UV-0ΔNLS vaccine elicited significant responses in wild-type but not μMT mice (Fig 7C). Splenocytes from live-0ΔNLS-vaccinated μMT mice contained twice as many IFN-γ-spot-forming cells relative to live-0ΔNLS-vaccinated wild-type mice (p<0.01). However, this difference only reflected the fact that T cells occur at twice the normal frequency in μMT splenocytes, because 60% of splenocytes in wild-type mice are B cells (data not shown). Collectively, the results suggested that B cells played an important role in priming T-cell responses to the UV-0ΔNLS vaccine, but were dispensable in generating robust CD8^+^ T-cell responses to the live-0ΔNLS vaccine.

### B-cell-deficient mice exhibit defective 0ΔNLS vaccine-induced protection against HSV-2

**Fig 8:**
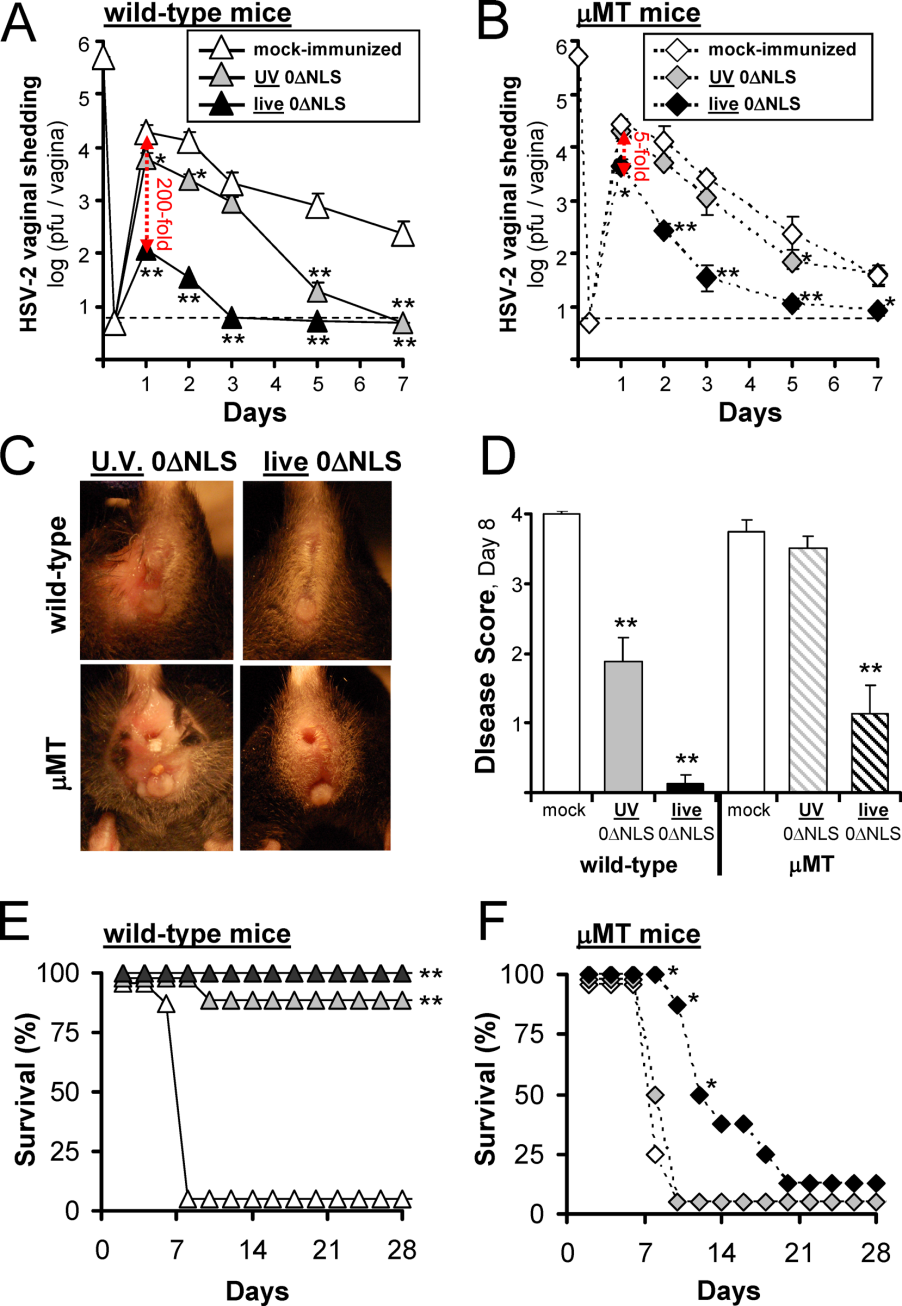
Loss of B-cell function in μMT mice correlates with impaired vaccine-induced protection against HSV-2. Mice were treated with 2 mg medoxyprogesterone 7 and 3 days prior to vaginal HSV-2 challenge. On Day 70, mock-, UV-0ΔNLS-, or live-0ΔNLS-immunized mice were challenged with 500,000 pfu per vagina of HSV-2 MS. **(A and B)** Mean ± sem HSV-2 MS shedding from the vaginas of **(A)** wild-type mice or **(B)** μMT mice at times post-challenge (n = 8 per group). The dashed line denotes the lower limit of detection of the plaque assay. **(C)** Representative examples of perivaginal disease on Day 8 post-challenge in wild-type or μMT mice immunized with the UV-0ΔNLS or live-0ΔNLS vaccines. **(D)** Mean ± sem of disease scores in mice on Day 8 post-challenge (n = 8 per group). **(E and F)** Survival frequencies for each group of **(E)** wild-type or **(F)** μMT mice are plotted as a function of time post-challenge. Regarding survival frequency, a single asterisk (*) denotes p < 0.05 and a double asterisk (**) denotes p < 0.001 that the frequency of survival in immunized mice was equivalent to mock-immunized control mice, as determined by Fisher's Exact Test. Regarding viral shedding data and disease scores, a single asterisk (*) denotes p < 0.05 and a double asterisk (**) denotes p < 0.001 that the indicated value was equivalent to mock-immunized control mice on that day, as determined by one-way ANOVA and Tukey's post-hoc-test.

Resistance to lethal HSV-2 vaginal challenge was compared in wild-type mice and B-cell-deficient mice that had been vaccinated with either the live- or UV-0ΔNLS vaccines. B-cell-deficient μMT mice exhibited a profound defect in early vaccine-induced control of HSV-2 challenge. Specifically, at 24 hours post-challenge, wild-type recipients of the live-0ΔNLS vaccine shed 200-fold less HSV-2 per vagina relative to mock-immunized controls (Fig 8A). In contrast, μMT recipients of the live-0ΔNLS vaccine shed only 5-fold less HSV-2 per vagina relative to mock-immunized controls (Fig 8B). Likewise, at 48 and 72 hours post-challenge, μMT recipients of the live-0ΔNLS vaccine continued to shed significantly more HSV-2 than wild-type recipients of the same vaccine (p<0.001, Fig 8A vs 8B). The efficacy of the UV-0ΔNLS vaccine was also decreased in μMT mice; wild-type mice that received the UV-0ΔNLS vaccine shed 40-fold less HSV-2 than mock-immunized controls at Days 5 and 7 (Fig 8A), whereas no such decrease was observed in UV-0ΔNLS-vaccinated μMT mice (Fig 8B).

The magnitude of vaginal HSV-2 shedding correlated with disease progression. Mock-immunized wild-type and μMT mice shed the highest levels of HSV-2, had the highest disease scores (Fig 8D), and succumbed to HSV-2 challenge within just 8 ± 1 days (Fig 8E and 8F). Wild-type mice immunized with the UV-0ΔNLS vaccine were only partially protected against death (Fig 8E) and experienced significant perivaginal disease (Fig 8C and 8D). The UV-0ΔNLS vaccine was not protective in μMT mice, and thus all of these mice developed lethal disease (Fig 8C, 8D and 8F). The live-0ΔNLS vaccine completely prevented perivaginal disease and death in wild-type mice (Fig 8C, 8D and 8E). In contrast, μMT recipients of the live-0ΔNLS vaccine experienced limited perivaginal disease (Fig 8C and 8D) and most succumbed to slowly progressive disease following HSV-2 challenge (Fig 8F). These results indicated that B cells were necessary for complete vaccine-induced protection against HSV-2 challenge. Since B cells were not required to prime CD8^+^ T-cell responses in recipients of the live-0ΔNLS vaccine, the results suggested that virus-specific antibodies were directly contributing to the observed protection.

### HSV-2 antiserum restores protection in live 0ΔNLS-vaccinated B-cell-deficient mice

**Fig 9:**
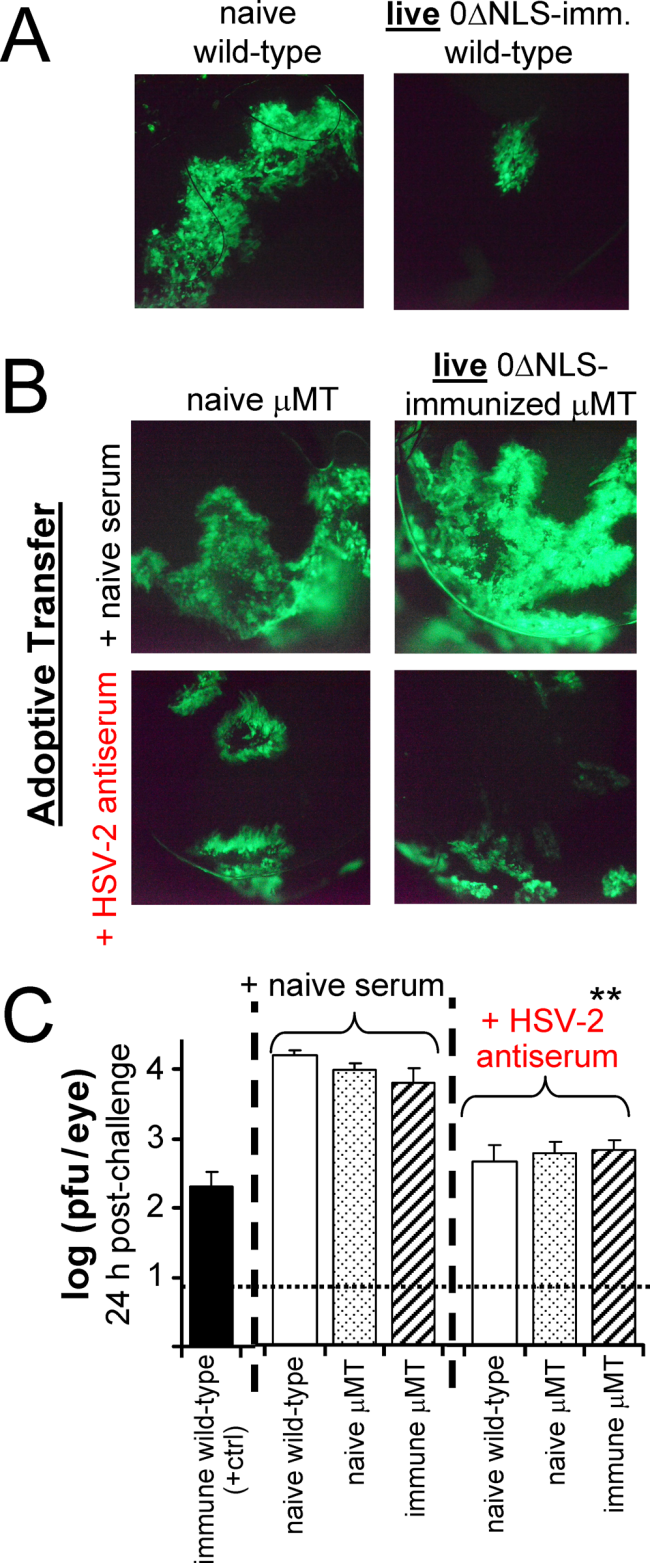
Passive transfer of HSV-2 antiserum restricts early HSV-2 MS-GFP spread in naïve mice and live-0ΔNLS-immunized μMT mice. On Day 70, naïve or live-0ΔNLS-immunized mice were challenged with 200,000 pfu per eye of HSV-2 MS-GFP. **(A)** GFP expression at 24 h post-challenge showing the extent of HSV-2 MS-GFP spread in representative corneas of naïve or live-0ΔNLS-immunized wild-type mice. **(B)** GFP expression at 24 h post-challenge showing the extent of HSV-2 MS-GFP spread in representative corneas of naïve or live-0ΔNLS-immunized μMT mice that were treated with naïve serum or HSV-2 antiserum. **(C)** HSV-2 MS-GFP shedding at 24 h post-challenge from the eyes of mice treated with naïve serum or HSV-2 antiserum (n = 5 per treatment group). The dashed line denotes the lower limit of detection of the plaque assay. The double asterisk (**) denotes p < 10^−6^ that viral shedding was equivalent in mice treated with naïve serum versus HSV-2 antiserum, as determined by a paired t-test.

To further investigate the role of HSV-2-specific antibodies in vaccine-induced protection, an experiment was conducted to determine if passive transfer of HSV-2-specific antibodies could be used to render live-0ΔNLS-immunized μMT mice fully resistant to HSV-2 challenge. Pooled serum was collected from wild-type recipients of the live-0ΔNLS vaccine, and was transferred by intraperitoneal injection to naïve wild-type mice, naïve μMT mice, and live-0ΔNLS-immunized μMT mice one day before and after ocular challenge with HSV-2 MS-GFP. At 24 hours post-challenge, GFP expression and HSV-2 MS-GFP spread in mouse corneas was assessed. As expected, wild-type recipients of the live-0ΔNLS vaccine exhibited a profound reduction in GFP expression relative to naïve wild-type mice (Fig 9A). Passive transfer of HSV-2 antiserum restricted the early spread of HSV-2 MS-GFP in the corneas of both naïve and live-0ΔNLS-immunized μMT mice relative to controls that received non-immune serum (Fig 9B). All groups of mice that received HSV-2 antiserum shed an average ~18-fold less virus per eye at 24 hours post-challenge relative to mice treated with non-immune serum (Fig 9C).

**Fig 10:**
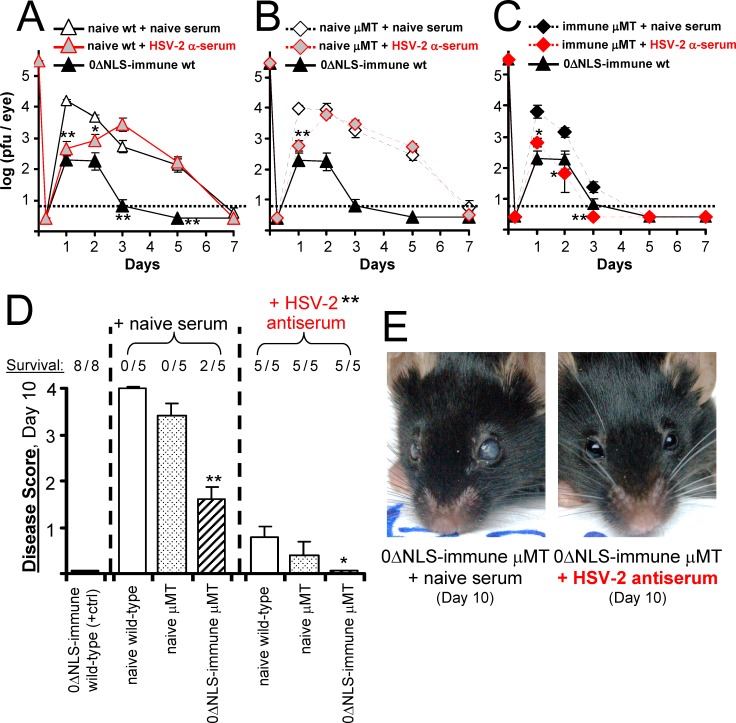
Passive transfer of HSV-2 antiserum restores complete vaccine-induced protection to live-0ΔNLS-immunized μMT mice. On Day 70, naïve or live-0ΔNLS-immunized wild-type and μMT mice were challenged with 200,000 pfu per eye of HSV-2 MS-GFP. **(A-C)** Mean ± sem of HSV-2 MS-GFP shedding at times post-challenge from **(A)** naïve wild-type (wt) mice, **(B)** naïve μMT mice, or **(C)** live 0ΔNLS-immunized μMT mice treated with naïve serum or HSV-2 antiserum (n = 5 per group). For reference, mean ± sem HSV-2 MS-GFP shedding in live-0ΔNLS-immunized wild-type mice is shown in panels A-C (n = 8; closed triangles). The dashed line denotes the lower limit of detection. A single asterisk (*) denotes p < 0.05 and a double asterisk (**) denotes p < 0.001 that HSV-2 MS-GFP shedding was equivalent on a given day in mice treated with HSV-2 antiserum versus naïve serum, as determined by a two-tailed t-test. **(D)** Mean ± sem of disease scores in naïve or live-0ΔNLS-immunized wild-type and μMT mice treated with naïve serum or HSV-2 antiserum on Day 10 post-challenge. **(E)** Representative ocular disease in live-0ΔNLS-immunized μMT mice treated with naïve versus HSV-2 antiserum.

The kinetics of ocular HSV-2 MS-GFP shedding was compared in all three groups of mice (Fig 10A–10C). Consistent with the results of a prior study from our lab [20], passive transfer of HSV-2 antiserum to naïve mice produced only a transient reduction in HSV-2 shedding at 24 hours post-challenge (Fig 10A and 10B). By 48 and 72 hours post-challenge, the capacity of HSV-2 antiserum to restrict HSV-2 MS-GFP shedding was negligible in naïve wild-type or μMT mice (Fig 10A and 10B). In contrast, passive transfer of HSV-2-specific antiserum to live-0ΔNLS-vaccinated μMT mice restored protection against HSV-2 MS-GFP shedding to levels that were statistically equivalent to live-0ΔNLS-vaccinated wild-type mice at all times post-challenge (Fig 10C). Likewise, all live-0ΔNLS-vaccinated μMT mice that received HSV-2 antiserum remained free of clinical signs and survived HSV-2 MS-GFP challenge (Fig 10D and 10E). In contrast, live-0ΔNLS-immunized μMT mice that received naïve serum all developed frank ocular disease (corneal opacity) between Days 8 and 10 post-challenge (Fig 10E), and 60% succumbed to a slowly progressing HSV-2 infection (Fig 10D). These results demonstrated that passive transfer of HSV-2-specific immune sera compensated for B-cell deficiency, thereby indicating that the primary function of the B cells in protection was the production of HSV-2-specific antibodies.

## Discussion

The experiments conducted herein elucidated two requirements for the live HSV-2 0ΔNLS vaccine to elicit complete protective immunity against HSV-2; namely the need for (1) active replication of the live vaccine and (2) a host B-cell response. UV-inactivation reduced the protective effects of the live-0ΔNLS vaccine, thus demonstrating that vaccine-induced protection was not solely attributable to antigens present in the inoculum. The limited *in vivo* replication of the attenuated vaccine may have contributed to protection by amplification of viral antigens, expression of viral antigens not present in virions, or both. Also, in the absence of adjuvants, replicating viruses are more likely than inactivated viruses to be recognized by innate sensors of infection such as toll-like receptors [44], gamma-interferon-inducible protein IFI-16 [45, 46], and retinoic acid-inducible gene 1 [47]. Such innate immune recognition elicits the co-stimulatory signaling necessary to initiate robust adaptive immune responses.

Interestingly, the UV-0ΔNLS vaccine elicited weak CD8^+^ T-cell responses in B-cell-deficient μMT mice relative to wild-type recipients of the UV-0ΔNLS vaccine (Fig 7). This finding indicates that B cells are important antigen presenting cells for CD8^+^ T responses to the UV-inactivated vaccine. In contrast, the live HSV-2 0ΔNLS vaccine appeared to elicit an equivalent CD8^+^ T-cell response in B-cell deficient μMT mice and wild-type mice. We postulate that the live-0ΔNLS vaccine's capacity for *de novo* antigen synthesis (Fig 1B and 1C) likely accounts for its increased efficacy at eliciting a CD8^+^ T-cell response in μMT mice [48, 49]. The experiments comparing HSV-2 vaccine performance in wild-type versus μMT mice demonstrated that B cells were required for full protection. Vaccination of B-cell-deficient μMT mice, even with the potent live HSV-2 0ΔNLS vaccine, failed to protect them from pathogenesis and lethal disease. These results are consistent with earlier experiments from Milligan and colleagues who, in 2000, demonstrated that μMT mice immunized with a live HSV-2 thymidine kinase^-^ mutant shed high titers of HSV-2 at early times post-challenge and remained more vulnerable to HSV-2 challenge than vaccinated wild-type mice [50].

### Virus-specific antibodies and T cells: two indispensable halves of protective immunity to HSV-2

A pivotal role for T cells in protective immunity to HSV emerged in the 1970s [51, 52] and by the 1990s, it became evident that the timing of T-cell infiltration into HSV-infected tissues precisely correlated with the non-cytolytic suppression of viral replication [53–60]. The evidence that T cells play a role in protective immunity to HSV-1 and HSV-2 is indisputable.

In the past decade, herpes immunologists have focused with great interest on studies of T-cell-mediated control of HSV infections. For example, many recent, influential studies in the field have been performed in experimental models where immunity to HSV was conferred upon naïve mice by adoptive transfer of pure populations of CD4^+^ and/or CD8^+^ T-cells specific for gD_315–327_ or gB_498-505_, respectively [61–66]. Likewise, there has been growing interest in T-cell-epitope-based HSV-2 vaccines that introduce recipients to HSV-2 epitopes embedded in carrier proteins, but which do not include B-cell antigens [67–73]. The Agenus HerpV vaccine is such a T-cell epitope-based HSV-2 vaccine that is currently in human clinical trials [74].

As immunologists have focused increasingly on the role of HSV-specific T cells in protective immunity, there has been a corresponding tendency to increasingly explain protective immunity to HSV as a primarily T-cell-mediated event [75–78]. However, the results of the current study provide a reminder that HSV-specific antibodies also have a critical role to play in protective immunity to HSV-2. In light of the available evidence, it would seem that the most effective HSV-2 vaccines will be those that elicit both a robust B- and T-cell response.

### Why don't virus-specific T cells confer complete protection against HSV-2?

Despite a robust T-cell response, live-0ΔNLS-immunized μMT mice were slow to control vaginal HSV-2 challenge. So, why don't virus-specific T cells alone offer complete protection? Following vaccination, virus-specific T-cell populations contract into a relatively small subset of memory cells that requires time to be activated and expand into effectors. In addition, while tissue-resident memory T cells (T_RM_) at the site of a local infection can be protective [62, 63], most memory T cells reside in the vasculature and lymphoid organs prior to an infectious insult, and must extravasate out of blood vessels before they can contribute to the protective immune response [55, 56]. Such T cell recruitment to sites of HSV infection may take several days so experimental strategies such as “prime and pull” have been developed to enhance the number of T_RM_ cells at common sites of infection such as the vagina [62, 63]. However, a more straightforward approach may be to use a vaccine that also elicits a virus-specific antibody response that may enhance the rate of T-cell recruitment to sites of HSV-2 challenge. Antigen-bound antibody complexes are potent initiators of the classical complement cascade, which generates split products such as C3a, C4a, and C5a (anaphylatoxins) whose natural function is to provide pro-inflammatory and chemokine-like signals that recruit WBCs to sites of antigen-antibody complex formation [79, 80]. Further studies will be required to determine whether complement fixation or other non-neutralizing antibody functions represent critical mechanisms by which virus-specific antibodies provide such strong protection against HSV-2.

### Conclusion

The results of the current study do not diminish the established importance of T cells in immunity to HSV-2 [56, 60, 81, 82]. However, vaccine-induced T-cell responses require time to be activated and delivered to sites of HSV-2 infection. In contrast, antibodies are pre-formed and present in mucosal secretions and the lymphatics that bathe epithelial cells, and are thus poised to restrict viral spread immediately upon exposure. Thus, vaccinated μMT hosts lacking antibodies are more likely to experience disease simply because HSV-2 infection may spread unchecked during the time required to deliver effector T cells to virus-infected tissues.

There is precedent for antibodies and T cells to function via non-redundant mechanisms, which act in synergy, to provide complete protection against viral infection [83]. Thus it would seem prudent to vaccinate in a way that best activates all components of the adaptive immune system rather than focusing on pure T-cell epitope vaccines or subunit vaccines that primarily elicit an antibody response. The live HSV-2 0ΔNLS vaccine described herein is attenuated in immunocompromised *rag2* ^-/-^ mice [15, 19], expresses a wide breadth of HSV-2 antigens [29], elicits a strong antibody and CD8^+^ T cell response, and confers robust protection against HSV-2. Based on its exceptional safety and efficacy profile, we conclude that HSV-2 0ΔNLS is a strong candidate for human vaccination, and offers a new opportunity to prevent HSV-2 genital herpes.

## Materials and Methods

### Ethics Statements

Mice were handled in accordance with the National Institutes of Health Guide for the Care and Use of Laboratory Animals and the American Veterinary Medical Association Guidelines for Euthanasia. This study was approved by the Institutional Animal Care and Use Committees of both Southern Illinois University School of Medicine and Rocky Mountain Laboratories.

### Cells and viruses

Vero and U2OS cells were obtained from the American Type Culture Collection (Manassas, VA). Vero and U2OS cells were propagated in Dulbecco’s Modified Eagle’s medium (DMEM) supplemented with 10% fetal bovine serum (FBS), 100 U/ml penicillin G, and 100 mg/ml streptomycin, hereafter referred to as “complete DMEM.” Wild-type HSV-2 MS (ATCC) and HSV-2 MS-GFP [15] were propagated and titered on Vero cells. The HSV-2 *ICP0* ^-^ mutant virus, HSV-2 0ΔNLS [15], was propagated and titered in U2OS cells. Ultraviolet (UV) inactivation of HSV-2 0ΔNLS was achieved by placing 1.5 ml of virus solution in a 60-mm dish in a Spectroline UV crosslinker, and delivering four successive cycles of 1 mJ/cm^2^ of radiation (i.e., instrument set to '9999' x 100 μJ/cm^2^) to achieve a total dose of 4 mJ/cm^2^.

### Animal studies

#### i. Immunization and HSV-2 challenge

Female C7BL/10 mice and B-cell-deficient μMT mice were obtained through Taconic Farms (Germantown, NY). On Day 0, mice were anesthetized by i.p. administration of xylazine (7 mg/kg) and ketamine (100 mg/kg), and were immunized via right, rear footpad injection of 50 μl containing ***i*.** complete DMEM (mock-immunized), ***ii*.** 2x10^6^ pfu HSV-2 0ΔNLS, or ***iii*.** an equivalent volume of UV-inactivated HSV-2 0ΔNLS. On Day 30, mice were similarly immunized in their left, rear footpad. Consistent with methods our laboratory has previously published [21], mice that received a HSV-2 vaginal challenge were pre-treated 7 and 3 days prior to inoculation with 2 mg medoxyprogesterone (Depo-Provera®, Pfizer Inc., New York), which increases the efficiency of vaginal infection [84]. Immediately prior to HSV-2 inoculation, mice were anesthetized by i.p. administration of xylazine (7 mg/kg) and ketamine (100 mg/kg). Ocular challenge of mice was performed by scarifying the left and right corneas with a 27-gauge needle, blotting tear film from the eyes with tissue paper, and by placing 4 μl complete DMEM containing 25,000 pfu / μl of HSV-2 MS-GFP on each scarified eye. For vaginal challenge with HSV-2 MS, the vagina was cleared of mucus by introducing the cotton end of a cotton-tipped applicator into the vagina. Upon removal of the cotton swab, a pipettor was used to deliver 20 μl complete DMEM containing 25,000 pfu / μl of HSV-2 MS into the vaginal vault.

#### ii. Measurement of infectious HSV-2 titers in secretions

Viral titers in ocular tear film or the vaginal secretions of mice were determined at times after inoculation by swabbing the eye with a cotton-tipped applicator or inserting a cotton-tipped applicator into the vaginal vault, and transferring the tip into 0.4 ml complete DMEM. Viral titers were determined by a 96-well plate plaque assay on Vero cells cultured in complete DMEM containing 0.5% methlycellulose. After two to three days of incubation in each plaque assay, cell monolayers were stained with a solution of 20% methanol and 0.1% crystal violet and plaques were counted.

#### iii. In vivo imaging of HSV-2 MS-GFP

Fluorescent photomicrographs of the eyes of mice inoculated with HSV-2 MS-GFP were obtained on a TE2000 inverted fluorescent microscope (Nikon Instruments) fitted with a DP72 digital camera (Olympus America). Mice were anesthetized by i.p. administration of xylazine (7 mg/kg) and ketamine (100 mg/kg) and placed on a clear petri dish to photograph their left and right eyes with a 4x objective.

#### iv. Disease scores

Pathogenesis was evaluated in immunization groups between 8 and 10 days post-challenge, and each animal was assigned a disease score between 0 to 4 based on the following criteria. A disease score of 0 was assigned to animals that were indistinguishable from uninfected animals. Disease scores of 1 or 2 were assigned if modest or overt, respectively, superficial pathogenesis was noted (e.g., perivaginal inflammation or ocular opacity). Disease scores of 3 were assigned to animals that were alive but exhibited constitutional symptoms suggesting that euthanasia might be required within 72 hours. Disease scores of 4 were assigned to animals who had already died or been euthanized.

### Flow cytometric analysis of peripheral CD8^+^ T-cells

Whole blood was collected from mice at Day 37 post-immunization (Day 7 post-boost) by retroorbital sinus bleed using heparinized Natelson blood collecting tubes (Fisher Scientific, Waltham, MA), and whole blood was immediately processed for flow cytometric analysis of peripheral white blood cells. To this end, 100 μl whole blood was initially depleted of RBCs by mixing with 300 μl 1.3% Dextran T-500 and allowing to settle for 30 minutes [85, 86]. The WBC-enriched supernatant was further depleted of RBCs by ammonium-chloride lysis, and peripheral WBCs were resuspended in a final volume of 50 μl PBS + 2% FBS + 20 μg / ml anti-CD16/32 (Fc-γ block) for immunofluorescent staining and flow cytometry. WBCs were stained with ***i*.** 1:300 FITC-conjugated mouse anti-CD11a (Biolegend, San Diego, CA); ***ii*.** 1:2,000 PE-conjugated anti-Thy1.2 (BD Biosciences, San Jose, CA); ***iii*.** 1:800 PerCP/Cy5.5 anti-mouse CD43 activation-associated glycoform (Biolegend); and ***iv*.** 1:400 APC anti-mouse CD8α (BD Biosciences). Following antibody staining, the frequency of activated CD8^+^ Thy1.2^+^ cells bearing elevated levels of CD11a and CD43 was analyzed by four-color flow cytometry in an Accuri C6 flow cytometer (Accuri Cytometers, Inc., Ann Arbor, MI). It should be noted that the kinetics of upregulation of CD11a and CD43 in CD8^+^ T cells was analyzed in pilot experiments on Days 4, 5, 6, 7, and 8 post-boost. On Days 4 and 5 post-boost, significant differences were not observed between mock and live HSV-2 0ΔNLS-vaccinated mice in terms of the frequency of CD8^+^ T cells bearing the CD11a^hi^ CD43^hi^ activated phenotype. On Days 6, 7, and 8 post-boost, live HSV-2 0ΔNLS-vaccinated mice exhibited elevated frequencies of CD8^+^ T cells bearing the CD11a^hi^ CD43^hi^ activated phenotype with maximum frequencies being observed on Day 7 post-boost.

### Measurement of HSV-2 antibody levels

Blood was collected from mice at Day 60 post-immunization by retroorbital sinus bleed using heparinized Natelson blood collecting tube (Fisher Scientific), and all serum samples collected for antibody analysis were frozen at -80°C until analyzed.

#### i. Measuring neutralizing antibody titer

A 4.3 μl aliquot of each serum sample was added to a single well in the top row of a microtiter plate containing 89 μl of complete DMEM to achieve an initial 1: 21 dilution. Serial 0.33-log dilutions were achieved by serial transfer of 43: 93 μl dilutions to the bottom of the plate. Guinea pig complement (Rockland Immunochemicals, Gilbertsville, PA) was diluted 1:50 in complete DMEM and combined with an equal volume of 3,500 pfu per ml HSV-2 MS-GFP. The HSV-2 neutralization assay was initiated by combining 50 μl of virus-complement mixture with each serum dilution (50 μl) and incubating at 37°C. After 1 hour, 100 μl of a suspension containing 3 x 10^6^ Vero cells per ml was added to each well, and microtiter plates were incubated for 30 hours to allow HSV-2 plaques to form, at which time GFP^+^ plaques formed by HSV-2 MS-GFP were enumerated using a fluorescent microscope. The HSV-2 neutralizing titer of each serum sample was considered to be the reciprocal of the largest serum dilution that reduced the number of HSV-2 MS-GFP plaques in Vero cell monolayers by at least 80%.

#### ii. Measuring HSV-2^+^ cell-specific: IgG (Total, IgG_1_, and IgG_2_)

Single-cell suspensions of a mixture of HSV-2^+^ cells and uninfected (UI) cells were generated, as follows. Ten 100-mm dishes were seeded with 8 x 10^6^ Vero cells per dish in complete DMEM, and five dishes were inoculated 6 hours later with 5 pfu per cell of HSV-2 MS. Cells were harvested 12 hours after inoculation by aspirating culture medium, adding 2 ml PBS + 5 mM ethylene diamine tetraacetic acid (EDTA) pH 8.0, and dispersing cells from dishes by trituration with a P-1000 pipettor. HSV-2^+^ cells in suspension were labeled with a green fluorophore by the addition of carboxyfluorescein diacetate, succinimidyl ester (CFSE; Anaspec, Fremont, CA) to a concentration of 1 μM and incubated for 10 minutes. Excess CFSE was rinsed from cells and cells were fixed in 18% formaldehyde for 20 minutes. Cells were centrifuged, supernatants decanted, and cells resuspended in 90% methanol for 10 minutes. Cells were rinsed and resuspended in PBS + 2% FBS (PBS-F), and cell clumps were removed by passage through a 40-μM nylon mesh cell strainer (BD Biosciences) followed by passage through a 25-gauge needle. Cell density in single-cell suspensions of UI Vero cells and CFSE-labeled HSV-2^+^ cells was determined, and UI cells and HSV-2^+^ cells were combined in a 1:1 ratio. These test cells were centrifuged and resuspended at a concentration of 1.25 x 10^6^ cells per ml in PBS-F-Ig block solution (i.e., PBS-F supplemented with 20 μg / ml each of donkey γ-globulin and goat γ-globulin; Jackson Immunoresearch Laboratories, Inc., West Grove, PA). Aliquots of test cells (200 μl; 250,000 cells) were placed in a 96-well U-bottom plate and 7 μl of 1:200 diluted serum was added to each cell suspension to achieve a net serum dilution of 1:6,000. Cells were incubated at room temperature for 2 hours, and primary antibody was removed by two, sequential centrifugations and rinses.

To enumerate the amount of total IgG antibody, IgG subclass 1, or IgG subclass 2 that specifically bound HSV-2^+^ cells (relative to UI cell background controls), test cells were secondarily incubated with 1:1,000 dilutions of APC-conjugated goat anti-mouse antibody specific for IgG_1+2a+2b+3_, IgG_1_, or IgG_2c_ (Jackson Immunoresearch #115-135-164, 115-135-205, and 115-135-208, respectively). After a 1-hour incubation, excess secondary antibody was removed by three, sequential PBS-F rinses. Cells were resuspended in a total volume of 130 μl PBS-F and analyzed by two-color flow cytometry in the FL1 and FL4 channels of an Accuri C6 flow cytometer (Accuri Cytometers, Inc.). HSV-2^+^ cell-specific IgG levels were calculated based on the difference in mean fluorescent intensity (ΔMFI) of 15,000 HSV-2^+^ cells versus 15,000 UI cells. Background fluorescence was defined as the average ΔMFI-value observed in cell suspensions incubated with naïve serum.

### IFN-γ ELISpot Analysis

White 96-well filter plates with 0.45 μm pore size hydrophobic PVDF membrane (EMD Millipore, Billerica, MA) were used for ELISPOT Assays, and were coated one day prior to use with a monoclonal anti-mouse IFN-γ capture antibody per the kit manufacturer's directions (eBioscience, San Diego, CA). Prior to WBC addition, IFN-γ capture antibody was removed and filter plates were blocked with complete RPMI-1640. Spleens were harvested from mock, UV-0ΔNLS, or live 0ΔNLS-immunized C57BL/10 or μMT mice at Day 7 post-boost (Day 37 of the experiment), and spleen WBCs were isolated by forcing spleen cells through a 40 μM nylon mesh followed by hypotonic lysis of RBCs with 0.16 M ammonium chloride. WBCs were resuspended in complete RPMI-1640, counted on a hemacytometer, and brought to a cell density of 2 x 10^6^ cells per ml for the ELIspot assay. SIINFEKL (OVA) and SSIEFARL (HSV-2 gB_498-505_) peptides were purchased from Sigma-Aldrich (St Louis, MO) and lyophilized peptides were resuspended at 20 μg/μl in dimethylsulfoxide (DMSO). ELIspot wells were 1oaded with 200 μl containing 4 μg/ml peptide and 2 x 10^5^ WBCs. Each combination of mouse WBCs and stimulator treatment was performed in triplicate. ELISpot plates were incubated at 37°C for 48 hours to allow for IFN-γ secretion from responding cells. After incubation, the frequency of IFN-γ^+^ secreting cells was visualized by removing cells, and incubating wells sequentially with a biotinylated IFN-γ-detection antibody, streptavidin-horseradish peroxidase, and the insoluble substrate 9-aminoethylcarbazole per the kit manufacturer's directions (eBioscience). After allowing 30 minutes for substrate conversion, wells were rinsed with water, plates were dried, and spots were visualized and counted in a CTL ImmunoSpot S6 Core Analyzer (CTL Analyzers, Shaker Heights, OH).

### Passive transfer of HSV-2 antiserum to C57BL/10 and μMT mice

Donor C57BL/10 mice were footpad-immunized with either culture medium (mock) or live HSV-2 0ΔNLS on Days 0 and 30. On Days 55 and 64, blood was collected from naïve (mock) or live 0ΔNLS-immunized mice by retroorbital sinus bleed, and mice were euthanized on Day 68 and a terminal bleed was collected. Naïve sera from all mock-immunized C57BL/10 mice were pooled, and HSV-2 antisera from all live 0ΔNLS-immunized C57BL/10 mice were pooled. Groups of n = 5 recipient mice received adoptive transfers of 0.25 ml pooled HSV-2 antiserum or naïve serum by i.p. administration one day before and one day after (Days -1 and +1) ocular challenge with HSV-2 MS-GFP.

### Statistical analysis

Unless otherwise specified, all values presented are the mean ± standard error of the mean (sem) of replicate samples. Viral titers were determined by microtiter plaque assay and were statistically analyzed on a logarithmic scale (e.g., log [pfu / vagina]). Infectious virus was not detectable in some ocular or vaginal swabs of well-immunized animals. In such events, the sample was assigned a value of 2.6 pfu per swab (i.e., one-third the lower-limit of detection of the assay), such that all samples could be analyzed on a logarithmic scale. All shedding data were statistically analyzed using logarithmic values. The significance of differences in multiple group comparisons was compared by one-way analysis of variance (ANOVA) followed by Tukey’s post hoc t-test using GraphPad Instat v3.10 software (GraphPad Software, Inc., La Jolla, CA). The significance of difference between two groups was performed using the "t-test assuming equal variances" function of Microsoft Excel. The significance of differences in survival frequency was determined by Fisher's Exact Test using freely available software (Ref. [87]; http://quantpsy.org/fisher/fisher.htm).
